# Supplementary material for: Dorsomedial and ventromedial prefrontal cortex lesions differentially impact social influence and temporal discounting
Source: PLoS Biol. 2025 Apr 28;23(4):e3003079. doi: 10.1371/journal.pbio.3003079 (PMC12036846; doi:10.1371/journal.pbio.3003079)
Supplement: S1 Text — (PDF) [file pbio.3003079.s001.pdf]

S1 Text

Temporal impulsivity and preference uncertainty do not depend on depression or apathy levels

As control analyses, separate analyses of covariance (ANCOVAs) including BDI and AMI scores were conducted to compare temporal impulsivity (*km*) between groups while controlling for levels of depression and apathy. Controlling for depression levels (main effect of group: $F_{(2,115)} = 5.84$, $p = 0.004$, η^2 [95% CI] = 0.09 [0.01 0.18]; effect of covariate BDI scores: $F_{(1,115)} = 0.47$, $p = 0.492$, η^2 [95% CI] = 0.004 [0.00 0.03]) and controlling for apathy levels (main effect of group: $F_{(2,115)} = 5.17$, $p = 0.007$, η^2 [95% CI] = 0.08 [0.006 0.17]; effect of covariate AMI scores: $F_{(1,115)} = 2.32$, $p = 0.130$, η^2 [95% CI] = 0.02 [0.00 0.07]) both did not change any of our results.

Additionally, separate ANCOVAs incorporating BDI and AMI scores were conducted to compare preference uncertainty (*ku*) between groups, adjusting for levels of depression and apathy. Controlling for levels of depression (main effect of group: $F_{(2,115)} = 6.95$, $p = 0.001$, η^2 [95% CI] = 0.11 [0.02 0.21]; effect of covariate BDI scores: $F_{(1,115)} = 10.65$, $p = 0.001$, η^2 [95% CI] = 0.08 [0.01 0.17]) and controlling for levels of apathy (main effect of group: $F_{(2,115)} = 9.00$, $p < 0.001$, η^2 [95% CI] = 0.14 [0.04 0.25]; effect of covariate AMI scores: $F_{(1,115)} = 4.14$, $p = 0.04$, η^2 [95% CI] = 0.03 [0.00 0.09]) both did not change any of our results related preference uncertainty.
