# Supplementary material for: Dorsomedial and ventromedial prefrontal cortex lesions differentially impact social influence and temporal discounting
Source: PLoS Biol. 2025 Apr 28;23(4):e3003079. doi: 10.1371/journal.pbio.3003079 (PMC12036846; doi:10.1371/journal.pbio.3003079)
Supplement: S1 Table — (PDF) [file pbio.3003079.s002.pdf]

**S1 Table**. *Summary of demographic variables for each group and linear regression.*

| Variable | HC mean [SD] | mPFC mean [SD] | LC mean [SD] | HC vs mPFC | LC vs mPFC |
|---|---|---|---|---|---|
| Age | 60.73 [16.37] | 56.88 [10.72] | 56.24 [11.09] | 0.20 | 0.88 |
| Education | 15.39 [2.33] | 12.91 [2.86] | 13.71 [2.78] | <0.001 | 0.29 |
| AMI | 1.15 [0.45] | 1.43 [0.60] | 1.36 [0.61] | 0.014 | 0.67 |
| BDI | 6.16 [8.50] | 10.09 [7.56] | 13.82 [10.87] | 0.033 | 0.15 |
| Trail A | 29.40 [11.22] | 27.00 [10.02] | 30.35 [14.26] | 0.33 | 0.33 |
| Trail B | 66.57 [26.00] | 77.19 [56.50] | 76.25 [34.94] | 0.19 | 0.93 |

Note. HC: healthy controls; LC: lesion controls; SD: standard deviation of the mean; Education: education years; AMI: Apathy-Motivation Index; BDI: Beck Depression Inventory; Trail A / B: Trail Making Test Part A / Part B; F: female; *p* values from simple linear regressions, with the reference level set to the mPFC group.
