# Supplementary material for: Dorsomedial and ventromedial prefrontal cortex lesions differentially impact social influence and temporal discounting
Source: PLoS Biol. 2025 Apr 28;23(4):e3003079. doi: 10.1371/journal.pbio.3003079 (PMC12036846; doi:10.1371/journal.pbio.3003079)
Supplement: S2 Table — (PDF) [file pbio.3003079.s003.pdf]

S2 Table. *Linear mixed-effects model predicting learning performances.*

**Fixed effect**	***beta***	**95% CI**	***t***	***p***	**BDI**	**AMI**
(Intercept)	79.78	[77.96 81.60]	86.33	<0.001	<0.001	<0.001
Group (*HC* vs *mPFC*)	3.22	[1.03 5.42]	2.90	0.004	0.004	0.013
Group (*LC* vs *mPFC*)	-0.66	[-3.71 2.38]	-0.43	0.668	0.562	0.628
Others (*patient* vs *impulsive*)	0.47	[-1.22 2.15]	0.55	0.584	0.626	0.633
Group (*HC* vs *mPFC*) x Others	1.68	[-0.34 3.71]	1.64	0.103	0.091	0.093
Group (*LC* vs *mPFC*) x Others	0.64	[-2.16 3.45]	0.45	0.651	0.639	0.615

Note. HC: healthy control group; mPFC: mPFC lesion group; LC: lesion control group; 95% CI: 95% confidence intervals. BDI: supplementary analysis controlling for participants' levels of depression (BDI scores) by including this as a fixed effect in the model (main effect of depression on learning accuracy p = 0.444). AMI: supplementary analysis controlling for participants' levels of apathy (AMI scores) by including this as a fixed effect in the model (main effect of apathy on learning accuracy p = 0.354). The mPFC lesion group as the reference group.
