# Supplementary material for: Dorsomedial and ventromedial prefrontal cortex lesions differentially impact social influence and temporal discounting
Source: PLoS Biol. 2025 Apr 28;23(4):e3003079. doi: 10.1371/journal.pbio.3003079 (PMC12036846; doi:10.1371/journal.pbio.3003079)
Supplement: S3 Table — (PDF) [file pbio.3003079.s004.pdf]

S3 Table. *Linear mixed-effects model predicting susceptibility to social influence, with self baseline temporal impulsivity as covariates (centred around the grand mean)*

**Fixed effect**	***beta***	**95% CI**	***t***	***p***	**BDI**	**AMI**
(Intercept)	0.53	[0.31 0.76]	4.63	<0.001	<0.001	<0.001
Group (*HC* vs *mPFC*)	-0.20	[-0.46 0.06]	-1.49	0.137	0.149	0.114
Group (*LC* vs *mPFC*)	-0.41	[-0.77 -0.05]	-2.24	0.026	0.018	0.023
Others (*patient* vs *impulsive*)	-0.21	[-0.44 0.01]	-1.88	0.061	0.053	0.050
Self baseline *km*	-0.09	[-0.23 0.05]	-1.28	0.204	0.155	0.202
Group (*HC* vs *mPFC*) x Others	0.28	[0.03 0.54]	2.17	0.031	0.027	0.026
Group (*LC* vs *mPFC*) x Others	0.06	[-0.30 0.41]	0.31	0.759	0.729	0.704
Group (*HC*) x Self baseline *km*	0.05	[-0.11 0.21]	0.58	0.565	0.540	0.629
Group (*LC*) x Self baseline km	0.08	[-0.13 0.30]	0.75	0.453	0.441	0.436
Others x Self baseline *km*	0.13	[-0.01 0.26]	1.88	0.062	0.059	0.056
Group (*HC*) x Others x Self baseline *km*	-0.09	[-0.24 0.07]	-1.08	0.281	0.305	0.319
Group (*LC*) x Others x Self baseline *km*	-0.07	[-0.28 0.14]	-0.63	0.531	0.523	0.511

Note. HC: healthy control group; mPFC: mPFC lesion group; LC: lesion control group; 95% CI: 95% confidence intervals. BDI: supplementary analysis controlling for participants' levels of depression (BDI scores) by including this as a fixed effect in the model (main effect of depression on signed KL divergence *p* = 0.451). AMI: supplementary analysis controlling for participants' levels of apathy (AMI scores) by including this as a fixed effect in the model (main effect of apathy on signed KL divergence *p* = 0.815). The mPFC lesion group as the reference group.
