# Supplementary material for: Dorsomedial and ventromedial prefrontal cortex lesions differentially impact social influence and temporal discounting
Source: PLoS Biol. 2025 Apr 28;23(4):e3003079. doi: 10.1371/journal.pbio.3003079 (PMC12036846; doi:10.1371/journal.pbio.3003079)
Supplement: S4 Table — (PDF) [file pbio.3003079.s005.pdf]

S4 Table. *LMM predicting susceptibility to social influence, with self baseline temporal impulsivity as covariates (centred around the grand mean), controlling for the order of others' preferences*

**Fixed effect**	***beta***	**95% CI**	***t***	***p***
(Intercept)	0.51	[0.26 0.76]	4.06	<0.001
Group (*HC* vs *mPFC*)	-0.20	[-0.46 0.06]	-1.49	0.137
Group (*LC* vs *mPFC*)	-0.41	[-0.77 -0.05]	-2.22	0.028
Others (*patient* vs *impulsive*)	-0.21	[-0.44 0.01]	-1.89	0.060
Self baseline *km*	-0.09	[-0.23 0.05]	-1.28	0.202
Order of others' preferences	0.04	[-0.17 0.24]	0.35	0.729
Group (*HC* vs *mPFC*) x Others	0.28	[0.03 0.54]	2.17	0.031
Group (*LC* vs *mPFC*) x Others	0.06	[-0.30 0.41]	0.31	0.757
Group (*HC*) x Self baseline *km*	0.05	[-0.11 0.21]	0.58	0.560
Group (*LC*) x Self baseline km	0.08	[-0.14 0.29]	0.69	0.488
Others x Self baseline *km*	0.13	[-0.01 0.26]	1.88	0.061
Group (*HC*) x Others x Self baseline *km*	-0.09	[-0.24 0.07]	-1.09	0.279
Group (*LC*) x Others x Self baseline *km*	-0.07	[-0.28 0.14]	-0.63	0.530

Note. HC: healthy control group; mPFC: mPFC lesion group; LC: lesion control group; 95% CI: 95% confidence intervals. The mPFC lesion group is the reference group.
