# Supplementary material for: Dorsomedial and ventromedial prefrontal cortex lesions differentially impact social influence and temporal discounting
Source: PLoS Biol. 2025 Apr 28;23(4):e3003079. doi: 10.1371/journal.pbio.3003079 (PMC12036846; doi:10.1371/journal.pbio.3003079)
Supplement: S5 Table — (PDF) [file pbio.3003079.s006.pdf]

S5 Table. *Correlations between impulsive and patient signed KL divergence (D_{KL}).*

	Overall	Healthy controls	mPFC lesions	Lesion controls
r_s	$r_{s(101)}$ = -0.13 [-0.32 0.06]	$r_{s(59)}$ = -0.09 [-0.33 0.17]	$r_{s(24)}$ = -0.16 [-0.51 0.25]	$r_{s(14)}$ = -0.22 [-0.65 0.31]
p	0.185	0.494	0.448	0.412
p (FDR)	0.494	0.494	0.494	0.494
BF_{01}	3.81	5.17	2.58	3.10

Note. $r_{s(df)}$: Spearman's Rho correlation coefficients (degrees of freedom); 95% confidence intervals are indicated in square brackets. *p*: p-values of correlations; *p* (FDR): false discovery rate (FDR)-corrected *p*-values. BF_{01} indicates the strength of evidence with Bayes factors contrasting the null hypothesis against the alternative hypothesis.
