# Supplementary material for: Dorsomedial and ventromedial prefrontal cortex lesions differentially impact social influence and temporal discounting
Source: PLoS Biol. 2025 Apr 28;23(4):e3003079. doi: 10.1371/journal.pbio.3003079 (PMC12036846; doi:10.1371/journal.pbio.3003079)
Supplement: S6 Table — (PDF) [file pbio.3003079.s007.pdf]

S6 Table. *LMM predicting susceptibility to social influence, with self baseline temporal impulsivity km as covariates (centred around the grand mean), controlling for self baseline preference uncertainty ku (centred around the grand mean)*

**Fixed effect**	***beta***	**95% CI**	***t***	***p***
(Intercept)	0.53	[0.31 0.76]	4.63	<0.001
Group (*HC* vs *mPFC*)	-0.21	[-0.48 0.05]	-1.60	0.111
Group (*LC* vs *mPFC*)	-0.36	[-0.73 0.02]	-1.88	0.062
Others (*patient* vs *impulsive*)	-0.21	[-0.43 0.02]	-1.84	0.068
Self baseline *km*	-0.09	[-0.23 0.05]	-1.30	0.194
Self baseline *ku*	-0.13	[-0.33 0.08]	-1.20	0.232
Group (*HC* vs *mPFC*) x Others	0.28	[0.02 0.54]	2.13	0.034
Group (*LC* vs *mPFC*) x Others	0.05	[-0.31 0.40]	0.27	0.787
Group (*HC*) x Self baseline *km*	0.05	[-0.11 0.21]	0.57	0.567
Group (*LC*) x Self baseline *km*	0.09	[-0.13 0.30]	0.82	0.415
Others x Self baseline *km*	0.13	[-0.01 0.26]	1.85	0.065
Group (*HC*) x Others x Self baseline *km*	-0.08	[-0.24 0.07]	-1.06	0.291
Group (*LC*) x Others x Self baseline *km*	-0.06	[-0.27 0.15]	-0.61	0.545

Note. HC: healthy control group; mPFC: mPFC lesion group; LC: lesion control group; 95% CI: 95% confidence intervals. The mPFC lesion group is the reference group.
