# Supplementary material for: Dorsomedial and ventromedial prefrontal cortex lesions differentially impact social influence and temporal discounting
Source: PLoS Biol. 2025 Apr 28;23(4):e3003079. doi: 10.1371/journal.pbio.3003079 (PMC12036846; doi:10.1371/journal.pbio.3003079)
Supplement: S7 Table — (PDF) [file pbio.3003079.s008.pdf]

S7 Table. *Correlations between learning performances and signed KL divergence (D_{KL}).*

	Healthy controls		mPFC lesions		Lesion controls	
	Impulsive	Patient	Impulsive	Patient	Impulsive	Patient
r_s	$r_{s(67)}$ = 0.03 [-0.21 0.26]	$r_{s(61)}$ = 0.06 [-0.19 0.30]	$r_{s(29)}$ = 0.17 [-0.20 0.49]	$r_{s(26)}$ = 0.28 [-0.11 0.59]	$r_{s(15)}$ = 0.13 [-0.37 0.58]	$r_{s(14)}$ = -0.11 [-0.57 0.41]
p	0.830	0.639	0.374	0.151	0.610	0.695
p (FDR)	0.830	0.830	0.830	0.830	0.830	0.830
BF_{01}	7.09	4.68	2.58	1.83	3.30	3.25

Note. $r_{s(df)}$: Spearman's Rho correlation coefficients (degrees of freedom); 95% confidence intervals are indicated in square brackets. *p*: *p*-values of correlations; *p* (FDR): false discovery rate (FDR)-corrected *p*-values. BF_{01} indicates the strength of evidence with Bayes factors contrasting the null hypothesis against the alternative hypothesis.
