# Supplementary material for: Dorsomedial and ventromedial prefrontal cortex lesions differentially impact social influence and temporal discounting
Source: PLoS Biol. 2025 Apr 28;23(4):e3003079. doi: 10.1371/journal.pbio.3003079 (PMC12036846; doi:10.1371/journal.pbio.3003079)
Supplement: S8 Table — (PDF) [file pbio.3003079.s009.pdf]

S8 Table. *Correlations between perceived similarity and signed KL divergence (D_{KL}).*

	Healthy controls		mPFC lesions		Lesion controls	
	Impulsive	Patient	Impulsive	Patient	Impulsive	Patient
r_s	$r_{s(67)}$ = 0.17 [-0.07 0.39]	$r_{s(61)}$ = 0.01 [-0.24 0.26]	$r_{s(28)}$ = 0.38 [0.02 0.65]	$r_{s(26)}$ = 0.40 [0.03 0.67]	$r_{s(15)}$ = -0.05 [-0.52 0.44]	$r_{s(14)}$ = -0.3 [-0.69 0.23]
p	0.169	0.948	0.041*	0.035*	0.848	0.254
p (FDR)	0.338	0.948	0.123	0.123	0.948	0.382
BF_{01}	3.85	6.18	0.60	0.54	3.64	1.59

Note. $r_{s(df)}$: Spearman's Rho correlation coefficients (degrees of freedom); 95% confidence intervals are indicated in square brackets. p: p-values of correlations; p (FDR): false discovery rate (FDR)-corrected p-values. BF_{01} indicates the strength of evidence with Bayes factors contrasting the null hypothesis against the alternative hypothesis. *$p < 0.05$.
