# Supplementary figures and images for: Dorsomedial and ventromedial prefrontal cortex lesions differentially impact social influence and temporal discounting
Source: PLoS Biol. 2025 Apr 28;23(4):e3003079. doi: 10.1371/journal.pbio.3003079 (PMC12036846; doi:10.1371/journal.pbio.3003079)

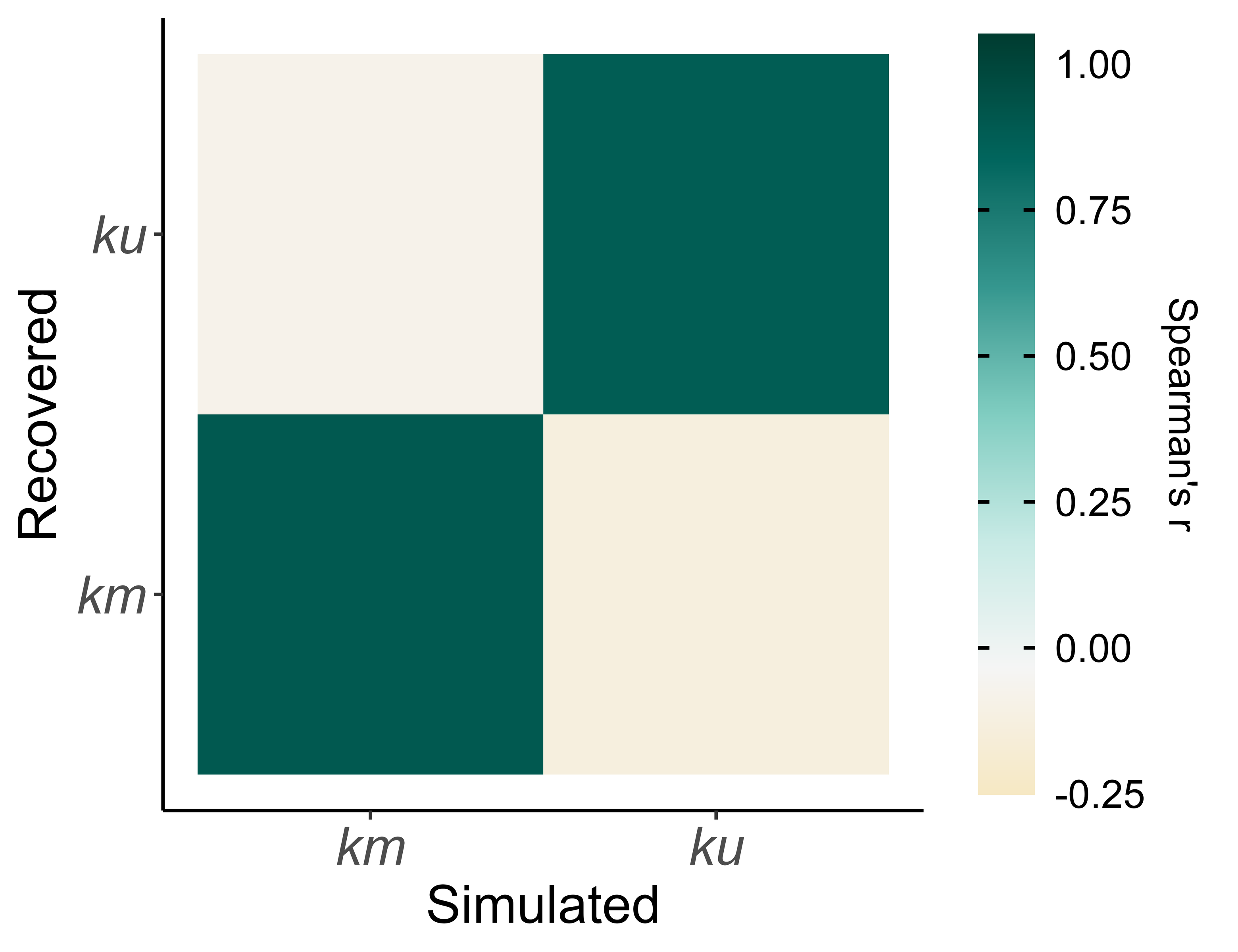

Supplement: S1 Fig — The confusion matrix illustrates Spearman’s Rho correlations between simulated and recovered (fitted) parameters. Both km and ku showed robust positive correlations between their true and recovered values, with all rs >0.87. (TIF) [file pbio.3003079.s010.tif]

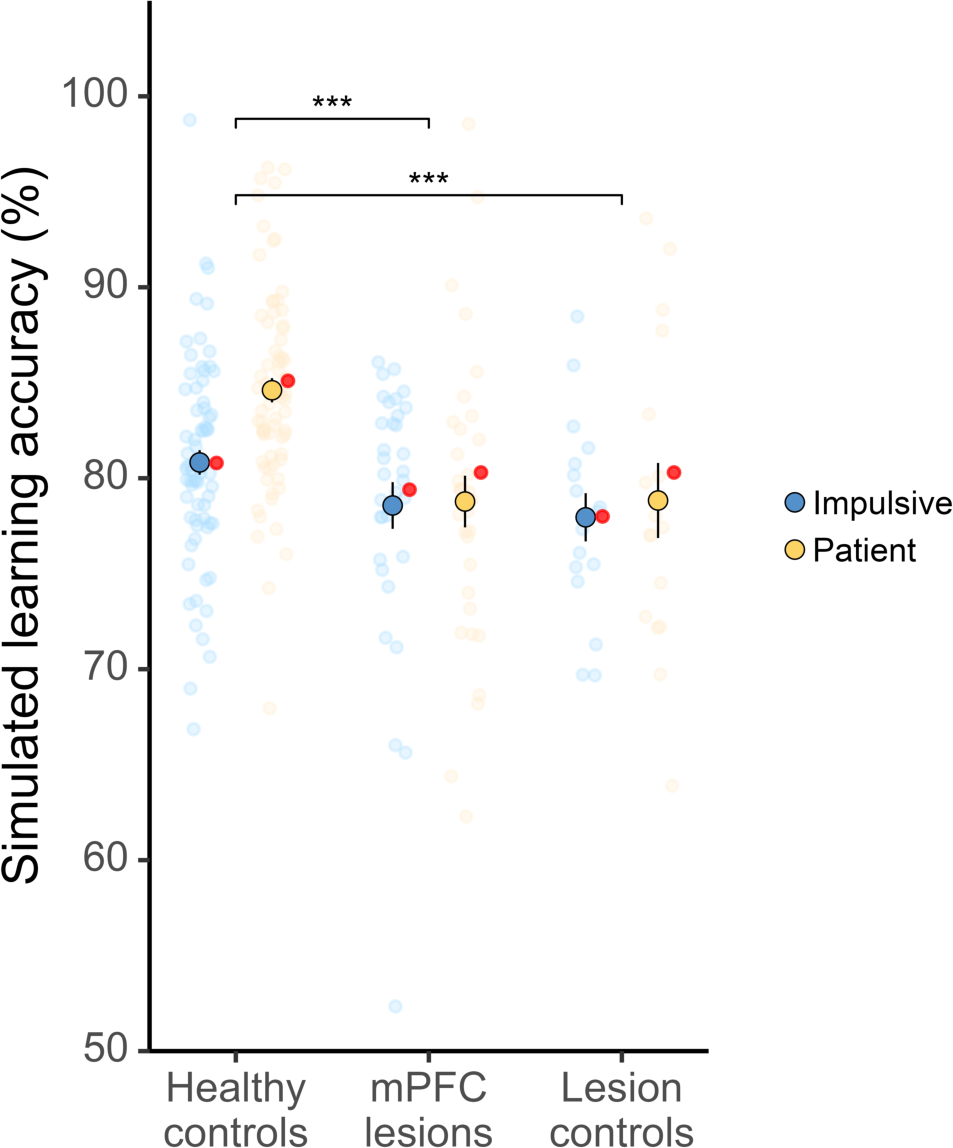

Supplement: S2 Fig — Posterior prediction replicates the key patterns observed in our empirical data. All three participant groups (healthy controls, mPFC lesions, and lesion controls) successfully learned the task (right-tailed exact binomial tests against 50%, all ps < 0.001). Compared to healthy controls, both mPFC lesion patients and lesion controls showed less accuracy in learning others’ preferences, regardless of whether these preferences were impulsive or patient (main effect mPFC vs. HC, b [95% CI] = −4.04 [−5.99 −2.09], p < 0.001; main effect LC vs. HC, b [95% CI] = −4.32 [−6.82 −1.83], p < 0.001). Participants generally performed better in terms of learning the preferences of patient others than impulsive ones (main effect patient vs. impulsive, b [95% CI] = 1.89 [0.95, 2.83], p < 0.001). Large bordered circles indicate the mean, error bars show the standard error of the mean, dots represent raw simulated data, and asterisks denote significant main effects of groups from the linear mixed-effects model. Note that the vertical axis starts at 50%, representing the chance level. **p < 0.001. Red dots are the means of actual data. (TIF) [file pbio.3003079.s011.tif]

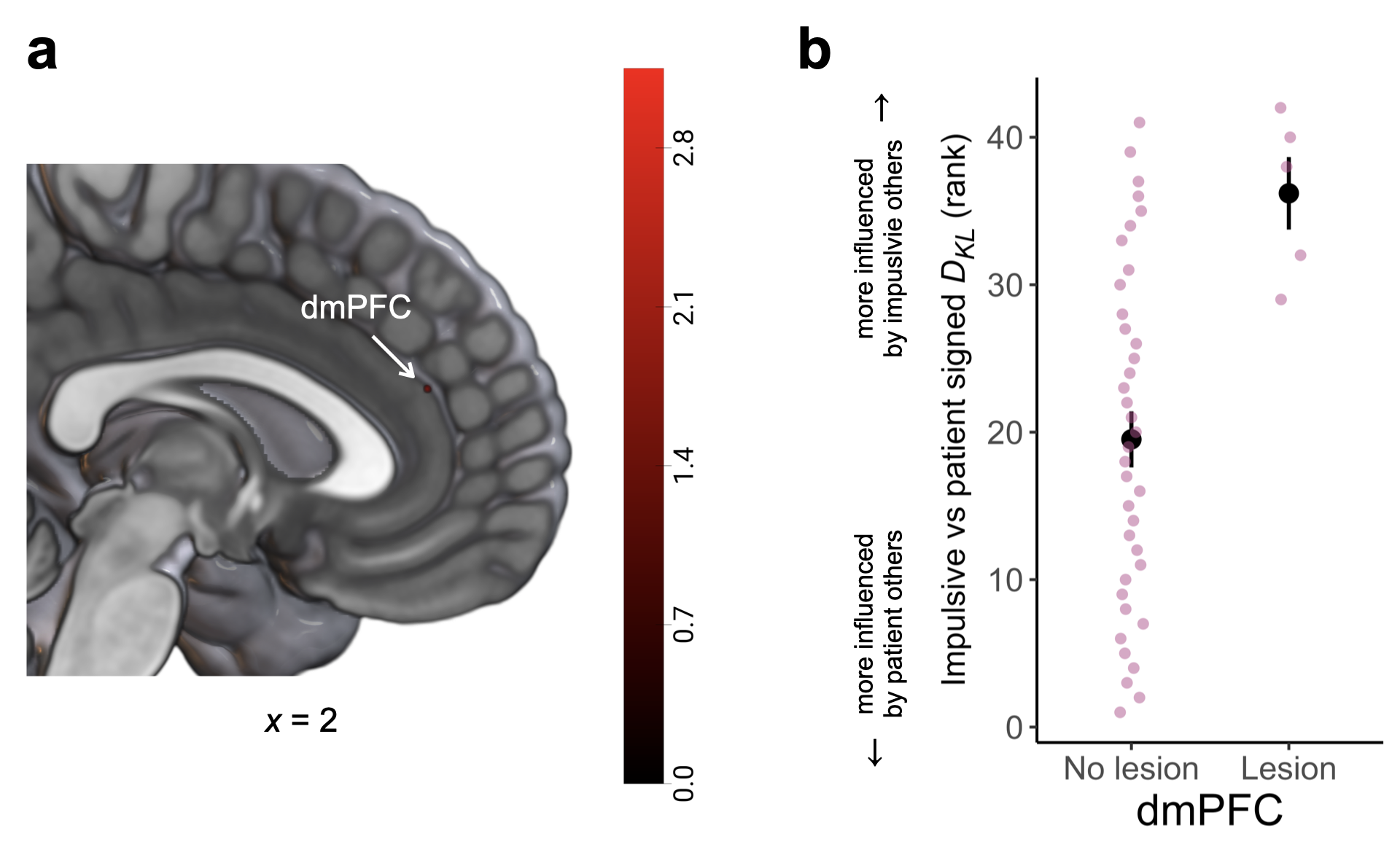

Supplement: S3 Fig — (a) Permutation-based, whole-brain, nonparametric voxel-based lesion-symptom mapping (VLSM) showed that damage to dorsomedial prefrontal cortex (dmPFC, area 9, peak MNI coordinate [±2, 40, 20]) was correlated with enhanced susceptibility to impulsive relative to patient social influence (permutation-based threshold free cluster enhancement (TFCE) p < 0.025). (b) Plotting the ranked contrasts between susceptibilities to impulsive and patient social influence, separately for participants with lesions or no lesion in this area identified by the VLSM analysis. N = 42 for this analysis where both patient and impulsive others were present. The underlying data and code used to generate this figure can be found at https://osf.io/qzurp/. Note: panel (B) is for illustrative purposes only and displays the ranked difference in signed KL divergence contrasts between participants with vs. without lesions, in the ROI defined by a wholebrain contrast. (TIF) [file pbio.3003079.s012.tif]

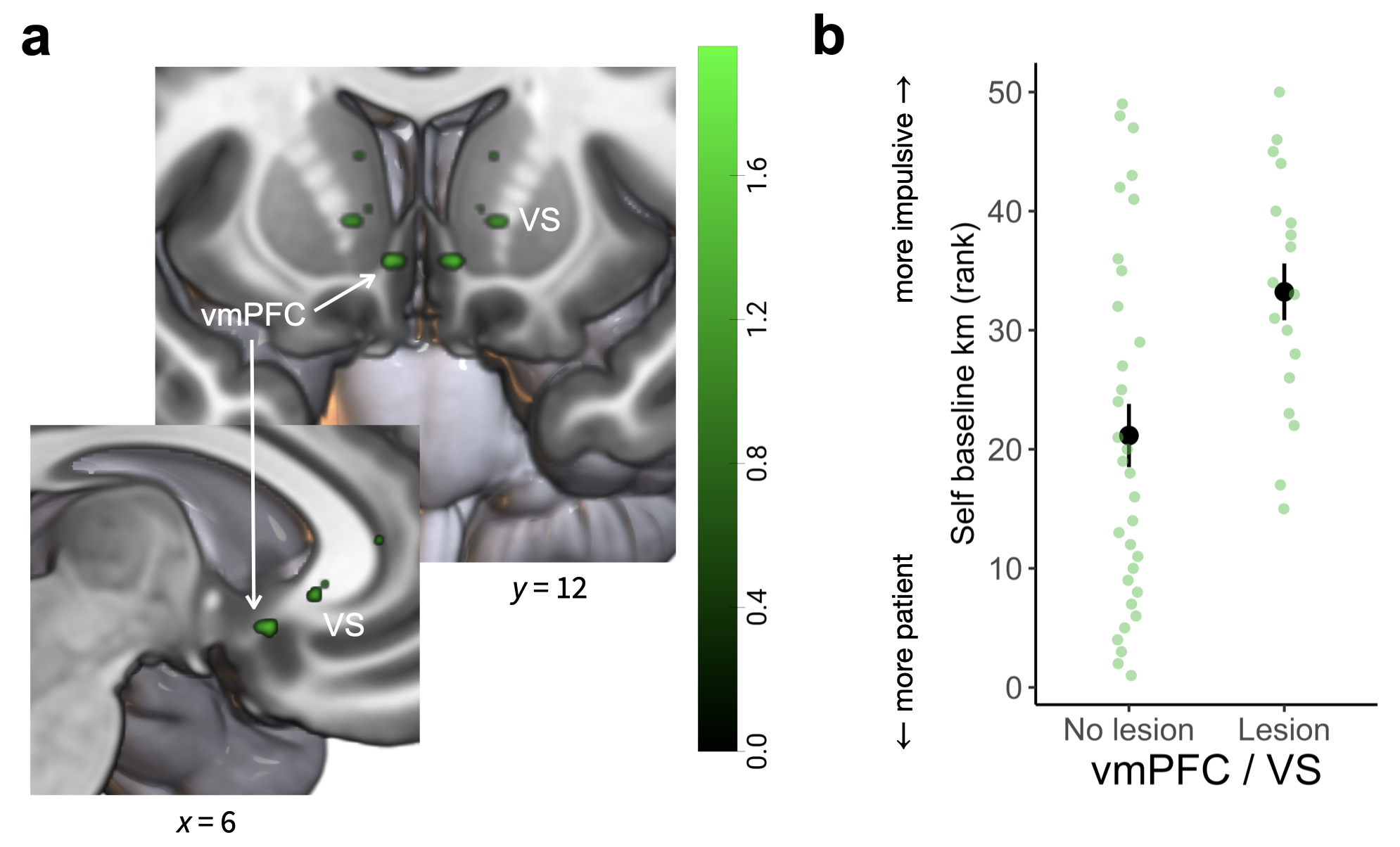

Supplement: S4 Fig — (a) Permutation-based, whole-brain, nonparametric voxel-based lesion-symptom mapping (VLSM) showed that the area 25 in the vmPFC as well as ventral striatum where damage was correlated with heightened temporal impulsivity (permutation-based threshold free cluster enhancement (TFCE) p < 0.05). (b) Plotting the ranked self baseline discounting preferences, 165 separately for participants with damage or no damage in the areas identified by the VLSM analysis (N 1= 50 in total). The underlying data and code used to generate this figure can be found at https://osf.io/qzurp/. Note: panel (B) is for illustrative purposes only and displays the ranked difference in self baseline discounting preferences between participants with vs. without lesions, in the ROI defined by a wholebrain contrast. (TIF) [file pbio.3003079.s013.tif]
