# Supplementary material for: Dorsomedial and ventromedial prefrontal cortex lesions differentially impact social influence and temporal discounting
Source: PLoS Biol. 2025 Apr 28;23(4):e3003079. doi: 10.1371/journal.pbio.3003079 (PMC12036846; doi:10.1371/journal.pbio.3003079)
Supplement: Attachment — Submitted filename: PLOS_Biol_social_influence_mPFC_reviewers_comments_final.pdf [file pbio.3003079.s017.pdf]

Reviewer #1 (Mark A. Thornton): The authors have very thoroughly addressed the issues I raised in my initial review, which were for the most part minor to begin with. I think this paper will make a substantial contribution to the literature in its present form.

Response: Thank you so much for taking the time to re-review our work and for providing us with such positive feedback. We really appreciate your time and effort helping us to improve the manuscript.

Reviewer #3: I thank the authors for their serious revision and constructive attitude. While I consider my concerns #3 and #4 satisfactorily addressed, I am not convinced by the arguments provided to rebut my concerns #1 and #2. Although it is not my intention to engage into a long and sterile back and forth revision process - let's agree to disagree -, let me restate -for the records- what I think are clear limitations that seriously question the value, robustness and generalizability of the claims proposed by the authors:

Response: Thank you so much for taking the time to re-review our work. We appreciate your additional feedback that has helped us improve the manuscript.

#1: I do not question the fact that participant do learn to adapt to the feedback that is proposed (about the fictitious participants' preference), but raise the (very credible IMHO) possibility that this learning (or a significant portion of it) can be done without mobilizing any social cognitive process. The same behavior could probably be observed if the task was to adjust to an explicit algorithm or bandit. The fact that the lab routinely engage in similar deceptive procedure seriously weaken the believability counter-argument. The incentivization argument is also not very strong, as participants are known to be very driven by intrinsic reward (solving a task, be correct) versus extrinsic rewards (meagre monetary bonuses). Hence, I sincerely doubt the very general claims about social influence.

Response: Thank you for your comments. We would like to reiterate some of the points we presented in our previous revision, that we believe directly address your remaining concerns. You highlight that 'The same behavior could probably be observed if the task was to adjust to an explicit algorithm or bandit'. However, this hypothesis was tested in a prior study using a similar paradigm to ours to measure social influence (Garvert et al., 2015, *Neuron*). The authors compared two conditions, one testing social influence as we have done in the current study, and also a control condition where participants were presented with identical stimuli and actions but were not required to simulate the preferences of other agents – whether human or computer (Garvert et al., 2015, *Neuron*). The design aimed to isolate and examine the specificity of social elements within the paradigm. They found that participants only changed their temporal discounting preferences when social simulation was engaged. This suggests that the ability to simulate others' mental states – a core aspect of social interaction (Boyd et al., 2011; Frith & Frith, 2023) – is essential for the observed changes in participants' preferences.

Second, regarding incentivisation, there was no 'correct' answer for participants' own preferences. While participants received feedback on the decisions they made for others, their performance in *Other* blocks had no impact on their bonus payments. Instead, it was their choices in *Self* blocks that determined their extra monetary reward, and in these blocks they received no correct/incorrect feedback. Therefore, the

changes in participants' temporal preferences cannot be explained by the claim that they were more motivated by intrinsic rewards (wanting to get the answer 'correct' over 'incorrect') compared to extrinsic ones (the monetary bonus payment). However, we appreciate that making these arguments more explicit would be beneficial for readers and have also included an additional limitation about the social/non-social nature of the task. We have edited our discussion section:

> ***Discussion**:*
> *The question of whether the brain has specialised regions and circuits for social behaviour is central to social neuroscience[8,83–85]. Previous work has identified how social specificity may be realised at different levels of explanation[24]. Our task had many features to enhance its ability to capture social processes, including two different social others with different preferences, informing participants that the choices they observed were from real others, and carefully probing for any disbelief in the social manipulation. Furthermore, existing studies including control conditions that match the same stimuli and actions, but do not require social simulation, failed to replicate changes in participants' discounting preferences[27]. This suggests that simulation of other agents' mental states – a central aspect of social interaction (Boyd et al., 2011; Frith & Frith, 2023) – is essential for the observed changes in people's preferencs, which highlights the importance of social component of the influence effect. To fully address whether shifts in people's own preferences occur in the absence of social influence, future studies could consider including a non-social control targeting different levels of explanation for social specificity. This additional control condition could reveal the cognitive boundaries and specific neural systems that underpin social influence and whether they are common or distinct from non-social processes.*

#2: I still think that the difference in the task performed by the subject is a serious problem in how we interpret the findings. Imagine a similar procedure in the realm of simple reinforcement-learning (RL) task. First, we want to control for the difference in how two groups value the outcome, and realize that Group 1 need 10£ to reach the same behavior as Group 2 with 1£ in a decision-making task. Then, the two groups perform a RL task (group 1 with 10£ rewards and Group 2 with 1£ rewards), and the authors report a significant difference in learning-rate between the groups: how can we draw a clear inference and interpretation of this finding ?

Response: Thank you for your query. Individual differences in certain behaviours within or between groups are inevitable, particularly in lesion studies where random assignment is not feasible. However, we ensured that the three groups were well-matched in demographic and neuropsychological measures. Additionally, we explicitly controlled for several covariates, including baseline temporal discounting rates, in our

statistical models to account for any baseline differences in temporal discounting. Furthermore, as previously discussed, the mPFC lesion group and the lesion control group exhibited similar baseline temporal discounting rates but differed in their response to social influence. It is important to note that our measures of susceptibility to social influence are evaluated relative to each participant's own baseline level of temporal discounting, providing a normalised reference point for comparison across individuals and hence across groups. Finally, our lesion analysis, which examines the effects of dmPFC damage on social influence, was conducted exclusively within the lesion groups that already had similar temporal discounting preferences, but brain damage in different locations. This provides clear evidence of the impact of dmPFC damage on social influence and further supports the findings from the group-level analysis. To acknowledge this we have added an additional note to the discussion to make our reasoning clearer:

> ***Discussion**:*
> *Whilst we did not observe any group differences between those with mPFC lesions and healthy controls in processing uncertainty at baseline, it would be interesting to evaluate the role of confidence in being influenced by other people. In contrast, healthy controls differed from both lesion groups in their baseline temporal discounting preferences. However, we controlled for baseline discounting preferences in our statistical models, and the two others that participants learnt about were modelled to be more impulsive or patient relative to participants' own baseline. The two lesion groups also did not differ, despite having brain damage in distinct areas. This ensured that differences in initial temporal discounting, before social influence, were accounted for.*
