# Supplementary material for: Dorsomedial and ventromedial prefrontal cortex lesions differentially impact social influence and temporal discounting
Source: PLoS Biol. 2025 Apr 28;23(4):e3003079. doi: 10.1371/journal.pbio.3003079 (PMC12036846; doi:10.1371/journal.pbio.3003079)
Supplement: Attachment — Submitted filename: SD_mPFC_response_to_reviewers_v5.docx [file pbio.3003079.s016.docx]

**Reviewer #1**: Su et al. present a lesion-based investigation of the role of medial prefrontal cortex in temporal impulsivity and (receiving) social influence.

Participants, including those with MPFC lesion, other lesions, and healthy controls, completed a temporal discounting task for themselves, and simulated more impulsive and more patient others. All groups of participants learned the idiosyncratic temporal preferences of both simulated others. Healthy controls were less impulsive than the lesion groups at baseline, and the non-MPFC lesion control group expressed less certain preferences at baseline. The MPFC lesion group demonstrated higher overall influence of the simulated others' preferences on their own subsequent intertemporal choices, with a particularly strong effect for the impulsive (vs. patient) other. VLSM identified a region in DMPFC associated with this effect. In contrast, lesions in VMPFC and the ventral striatum were associated with higher baseline impulsivity. Together, these results implicate MPFC - and in particular, DMPFC - in receptivity to social influence.

Generally, I think this is a strong paper, featuring a well-designed paradigm, sensible analyses, and an interesting research question, and clear writing. Below I list a few questions and comments I hope might improve it further:

**Response**: Thank you so much for your positive feedback on our work. We sincerely appreciate the time you invested in reviewing our work and providing valuable comments, which have greatly contributed to improving our manuscript.

**R1.1**: Perhaps I am misunderstanding, but in figures 5 and 6, I am concerned that the "b" panels are non-independent. That is, they show the ranked difference in KL divergence/baseline impulsivity between participants with vs. without lesions in the ROI defined by a wholebrain contrast. I realize that these panels do not reflect an inferential analysis, but this kind of circularity still exaggerates the size of the effect.

**Response**: Thank you for your query. You are correct that figures 5/6 panel b) show the ranked difference in signed KL divergence contrasts/baseline impulsivity between participants with vs without lesions in the ROI defined by a wholebrain contrast. We included these panels so that it is clear to see the group difference and the number of participants that contribute to the result, rather than performing additional inferential analysis by comparing the individual data points. Since not all participants necessarily have a lesion in the ROI or show the same pattern as the group, we believe these figures are still informative for easily understanding the nature and direction of the effects. To avoid confusion, we realise it would be helpful for readers to make the illustrative-only purposes clearer in the figure legends and have updated the legends accordingly:

***Figure 5 legend***:

***(b)*** *Plotting the ranked contrasts between susceptibilities to impulsive and patient social influence, separately for participants with damage or no damage in the areas identified by the VLSM analysis. N = 26 for this analysis where data from patient and impulsive was present. Note: panel (b) is for illustrative purposes only and displays the ranked difference in signed KL divergence contrasts between participants with vs. without lesions, in the ROI defined by a wholebrain contrast.*

***Figure 6 legend***:

***(b)*** *Plotting the ranked self baseline discounting preferences, separately for participants with damage or no damage in the areas identified by the VLSM analysis (N = 33). Note: panel (b) is for illustrative purposes only and displays the ranked difference in self baseline discounting preferences between participants with vs. without lesions, in the ROI defined by a wholebrain contrast.*

**R1.2**: Why don't the linear mixed effects models include any random slopes? As currently written, the models assume that the size of the patient vs. impulsive effect on KL divergence is the same across participants in the same group, which isn't necessarily true.

**Response**: We did not include random slopes in our linear mixed effects models since each of our participants only has one data point per condition (signed KL divergence and temporal impulsivity are calculated as single parameter values). Therefore, there is no within-participant variation which would be necessary for the inclusion of random slopes. For this reason, we did not include any random slopes for different others’ preferences, but we did include random intercepts to account for differences between participants. We have made this clearer in the revised manuscript:

***Supplementary Methods***

***Statistical analysis:***

*Note that each participant contributed a single parameter data-point and therefore these models could not contain random slopes.*

**R1.3**: Eyeballing figure 4, it looks like there might be a negative correlation across participants within the same group, such that those who are more influenced by the impulsive other are less influenced by the patient other, and vice versa. Is that true? And if it is, is that a substantive effect, or is it just something like an order effect?

**Response**: Thank you for this query. We have now run correlations between impulsive and patient signed KL divergence across all participants and within each group. No significant correlations were found in any condition, with Bayesian evidence (Schmalz et al., 2023) ranging from anecdotal to substantial supporting the null hypothesis (all BF_01_s > 2.58; new **Table S5,** reproduced below). In addition, controlling for the order of others’ preferences in our linear mixed-effects model did not change our results (see **Table S4** in the original supplement).

| **Table S5**. *Correlations between impulsive and patient signed KL divergence (D_KL_).* | | | | |
| --- | --- | --- | --- | --- |
|  | Overall | Healthy controls | mPFC lesions | Lesion controls |
| *r_s_* | *r_s(101)_* = -0.13  [-0.32 0.06] | *r_s(59)_* = -0.09  [-0.33 0.17] | *r_s(24)_* = -0.16  [-0.51 0.25] | *r_s(14)_* = -0.22  [-0.65 0.31] |
| *p* | 0.185 | 0.494 | 0.448 | 0.412 |
| *p* (FDR) | 0.494 | 0.494 | 0.494 | 0.494 |
| BF_01_ | 3.81 | 5.17 | 2.58 | 3.10 |

Note. r*_s(df)_* : Spearman’s Rho correlation coefficients (degrees of freedom); 95% confidence intervals are indicated in square brackets. *p*: p-values of correlations; *p* (FDR): false discovery rate (FDR)-corrected *p*-values. BF_01_ indicates the strength of evidence with Bayes factors contrasting the null hypothesis against the alternative hypothesis.

| **Table S4.** *LMM predicting susceptibility to social influence, with self baseline temporal impulsivity as covariates (centred around the grand mean), controlling for the order of others’ preferences* | | | | |
| --- | --- | --- | --- | --- |
| **Fixed effect** | ***beta*** | **95% CI** | ***t*** | ***p*** |
| (Intercept) | 0.51 | [0.26 0.76] | 4.06 | <0.001 |
| Group (*HC* vs *mPFC*) | -0.20 | [-0.46 0.06] | -1.49 | 0.137 |
| Group (*LC* vs *mPFC*) | -0.41 | [-0.77 -0.05] | -2.22 | 0.028 |
| Others (*patient* vs *impulsive*) | -0.21 | [-0.44 0.01] | -1.89 | 0.060 |
| Self baseline *km* | -0.09 | [-0.23 0.05] | -1.28 | 0.202 |
| Order of others’ preferences | 0.04 | [-0.17 0.24] | 0.35 | 0.729 |
| Group (*HC* vs *mPFC*) x Others | 0.28 | [0.03 0.54] | 2.17 | 0.031 |
| Group (*LC* vs *mPFC*) x Others | 0.06 | [-0.30 0.41] | 0.31 | 0.757 |
| Group (*HC*) x Self baseline *km* | 0.05 | [-0.11 0.21] | 0.58 | 0.560 |
| Group (*LC*) x Self baseline km | 0.08 | [-0.14 0.29] | 0.69 | 0.488 |
| Others x Self baseline *km* | 0.13 | [-0.01 0.26] | 1.88 | 0.061 |
| Group (*HC*) x Others x Self  baseline *km* | -0.09 | [-0.24 0.07] | -1.09 | 0.279 |
| Group (*LC*) x Others x Self  baseline *km* | -0.07 | [-0.28 0.14] | -0.63 | 0.530 |

Note. HC: healthy control group; mPFC: mPFC lesion group; LC: lesion control group; 95% CI: 95% confidence intervals. The mPFC lesion group is the reference group.

R1.4: While I agree that lesion evidence offers strong evidence for causation than neuroimaging, the lesions are obviously not randomly assigned, so the causal identification that they license isn't absolute. For example, if hypertension and impulsivity share a common cause, which doesn't seem impossible, then this could account for the association between lesions and baseline impulsivity through an alternative causal pathway.

**Response**: Thank you so much for raising this interesting and important point. We agree that whilst lesion studies offer advantages over neuroimaging there may still be alternative contributing factors for associations. Nonetheless, we would also emphasise that the presence of the lesion control group (who had the same pathology but in a different brain area) was designed to control for effects that simply correlate with having brain lesions. Moreover, our choice of patients who predominantly had aneurysmal haemorrhage – which unlike ischaemic infarcts - are stochastic events with relatively weak causal associations with impulsivity – also reduces the chance of a confounding variable. We have added this reasoning as a limitation and future direction in our discussion:

***Discussion****:*

*In addition to these novel findings, our study also has limitations. … Whilst we did not observe any group differences between those with mPFC lesions and healthy controls in processing uncertainty, it would be interesting to evaluate the role of confidence in being influenced by other people. Additionally, while we have used advanced lesion-symptom mapping with a relatively large cohort of patients to establish a causal link between the mPFC and susceptibility to social influence, there could be alternative explanations for some associations. For example, there could be shared causes that make brain lesions and impulsivity more likely to co-occur. However, the lesion control group was designed to control for effects that simply correlate with having brain lesions. Moreover, our choices of patients who predominantly had aneurysmal haemorrhages, which are stochastic events with relatively weak causal associations with impulsivity, also reduced the chance of a confounding variable influencing our findings. Future research would ideally take a multi-centre longitudinal approach to be able to provide even stronger causal evidence.*

**R1.5**: It is interesting that the impulsive others had a bigger influence on the lesion participants, given that all groups of participants learned more accurately about the patient others. Was there any association between participants' learning accuracy and the degree to which they were influenced by the others?

**Response**: Thank you for this query. Our correlational analyses did not find any significant correlations between participants’ learning accuracy and the extent to which they were influenced by others (see **Table S5** in the original supplement/**Table S7** in the revised supplement).

| **Table S7**. *Correlations between learning performances and signed KL divergence (D_KL_).* | | | | | | |
| --- | --- | --- | --- | --- | --- | --- |
|  | Healthy controls | | mPFC lesions | | Lesion controls | |
|  | Impulsive | Patient | Impulsive | Patient | Impulsive | Patient |
| *r_s_* | *r_s(67)_* = 0.03  [-0.21 0.26] | *r_s(61)_* = 0.06  [-0.19 0.30] | *r_s(29)_* = 0.17  [-0.20 0.49] | *r_s(26)_* = 0.28  [-0.11 0.59] | *r_s(15)_* = 0.13  [-0.37 0.58] | *r_s(14)_* = -0.11  [-0.57 0.41] |
| *p* | 0.830 | 0.639 | 0.374 | 0.151 | 0.610 | 0.695 |
| *p* (FDR) | 0.830 | 0.830 | 0.830 | 0.830 | 0.830 | 0.830 |
| BF_01_ | 7.09 | 4.68 | 2.58 | 1.83 | 3.30 | 3.25 |

Note. *r_s(df)_*: Spearman’s Rho correlation coefficients (degrees of freedom); 95% confidence intervals are indicated in square brackets. *p*: *p*-values of correlations; *p* (FDR): false discovery rate (FDR)-corrected *p*-values. BF_01_ indicates the strength of evidence for the null hypothesis.

**R1.6**: I could not directly review the data/code. The statement before the beginning of the manuscript states that they are fully available, but instead of an actual link, there is just a placeholder text reading "[URL]".

**Response**: Apologies. The data and code are now accessible through this link to the OSF project: <https://osf.io/qzurp/?view_only=a3ec8260fc684f85941f59023dd17126>.

***Data Availability Statement****:*

*Data and code for modelling and analysis are openly available at the open science framework (OSF):* [*https://osf.io/qzurp/*](https://osf.io/qzurp/)*.*

**Reviewer #2**: In this paper, the authors leveraged access to a relatively large sample of patients with damage to the mPFC to examine how the structural integrity of different regions within mPFC relate to temporal discounting, as well as to social influence. The authors replicated the finding that mPFC damage leads to increased discounting, but they built on this finding by pinpointing the exact (ventral) subregions associated with increased discounting. The authors also found novel evidence that the ventral striatum may play a causal role in future-oriented choice, although they do emphasize the exploratory nature of that finding. Finally, the authors provide compelling evidence that the dorsal portion of the mPFC is causally involved in maintaining one's own preferences as separate from another person's. While I'm less familiar with the literature on social influence, I believe that this is a pretty novel finding as well.

I really enjoyed this paper, so I don't have many comments. The analyses are sound, the results are interesting, and the writing is very clear. I just have a few small suggestions:

**Response**: Thank you so much for your positive feedback on our work. We sincerely appreciate the time you invested in reviewing our work and providing valuable comments, which have greatly contributed to improving our manuscript.

**R2.1**: The authors do a nice job in the Discussion speculating about why dmPFC damage might lead to increased susceptibility to social influence. I think the hypothesis that the dmPFC maintains a self-other distinction in some way makes sense. However, there are a couple of findings related to this that I would have wanted to see discussed as well. First, why do the authors think that the social influence results are driven by the impulsive condition rather than the patient condition? Could the authors comment on this asymmetry in the direction of social influence? Second, the authors bring up that their results might have to do with social learning, but they also found that there was no evidence that learning was associated with the extent of social influence. It's worth mentioning that null finding there as a caveat.

**Response**: Thank you for the opportunity to elaborate on our findings. We were also intrigued that there was a stronger influence in the impulsive compared to the patient condition, despite learning accuracy being not-significantly different, or ratings of similarity to patient and impulsive others. We can only speculate on the reason for this result as these additional control analyses do not reveal why there was an asymmetry. One possible explanation is that although the mPFC group did not report feeling more similar to the impulsive others, implicitly they were more swayed by others they perceive to behave as more similar to themselves. This aligns with the hypothesis that the dmPFC maintains the self-other distinction, as its lesioning made the mPFC group even more susceptible to the impulsive influence of others.

Regarding the association with social learning, in our task participants needed a high level of learning accuracy of others’ preferences in order to be influenced by them. All groups learnt with accuracy much greater than 50% (see results), which is different from many reinforcement learning studies where accuracy, particularly in older adults, is often closer to chance level (e.g., Cutler et al., 2021). Therefore, our study was not optimised in its design to directly address the social learning aspect, although we still feel it useful to draw on previous research on dmPFC involvement in learning social information to help interpret our findings in the broader literature. We suggest that other paradigms specifically designed to assess the effects of dmPFC on social learning, such as during mentalising or prosocial behaviour, would be useful in future research. We have added these two lines of reasoning to our discussion.

***Discussion****:*

*Therefore, one possibility is that damage to dmPFC could blur the self-other distinction and hinder the effective use of social information, prompting people to excessively depend on others for information, thereby increasing susceptibility to social influence. This process may drive the asymmetry we observed in susceptibility to impulsive vs patient social influence in those with dmPFC damage. Although they did not report feeling more similar to impulsive others, their similarity to impulsive others could drive an implicit process where they were particularly susceptible to being influenced by others who also displayed impulsive choices.*

*Therefore, given the central role of the dmPFC in reinforcement learning within social contexts, damage to the dmPFC may result in atypical social prediction errors which heighten social conformity. Future studies could probe these alternative explanations further. Notably, learning accuracy of others’ preference was intentionally high in the current paradigm to ensure all groups were able to learn others’ preferences so they could be influenced by them. Future paradigms could explicitly measure the effects of dmPFC damage on social learning in paradigms where learning accuracy is more variable such as whilst assessing mentalising^80^, vicarious learning^13,68,81^, or other social behaviours.*

**R2.2**: In line 267, the authors mention that participants with mPFC lesions "did not report feeling more similar to impulsive others." When I first read this (having not read the Methods section beforehand), I was confused about what this meant. Could the authors add a sentence to the first part of the Results section (and/or to the caption of Figure 2) just mentioning that the participants were also asked about their perceived similarity to the other choosers?

**Response**: We agree this would be helpful. We have added a description of the self-report measures in the first paragraph of the Results section.

***Results*:**

*All participants first engaged in an inter-temporal choice task to assess their baseline individual discounting preferences. Following this, they were introduced to the preferences of two other players who they were informed had participated in the same temporal discounting task previously. … They also completed a series of neuropsychological tests, self-report measures of depression and apathy, and self-reported their perceived similarity to both impulsive and patient others at the end of the experiment. The three groups were closely matched, displaying no significant differences in terms of age, gender, visual attention, and executive function. Additionally, the two lesion groups showed no differences between each other in education, depression, or apathy (see Methods and Table S1). Controlling for depression or apathy did not change any of our key results regarding group differences in temporal discounting or susceptibility to social influence (Supplementary Note 1, Supplementary Tables S2-S3).*

**R2.3**: I know that examining preference uncertainty was not the main goal of the study, but I'm intrigued by the finding that the participants with lesions outside the mPFC had more preference uncertainty. Would the authors consider doing an exploratory VLSM analysis to see where in the brain more damage is associated with more preference uncertainty?

**Response**: Thank you for your suggestion. The voxel-based lesion-symptom mapping (VLSM) analysis was restricted to voxels with lesions present in at least five participants, to reduce the risk of biased regression parameter estimates (Baldo et al., 2018). The lesion control group contained patients with lesions anywhere outside of mPFC, yet there was minimal overlap of lesioned voxels with no additional brain areas where at least 5 patients had overlapping damage (**Figure 1** in the original manuscript). This means that unfortunately we are not powered to assess the neural basis of greater preference uncertainty observed behaviourally in the lesion control sample.

**R2.4**: The authors (appropriately) control for baseline temporal discounting in their models that examine social influence. However, one could argue that controlling for baseline preference uncertainty is also important, in light of the fact that the LC group showed higher preference uncertainty than the other groups. Preference uncertainty also seems like it might be related to susceptibility to social influence; I could imagine that people whose preferences are more flexible or uncertain might be more susceptible. Could the authors run an additional analysis examining social influence that includes preference uncertainty as a covariate?

**Response**: Thank you for your suggestion. We have run an additional exploratory LMM including baseline preference uncertainty *ku* (continuous covariates, centred around the grand mean) (**Table S6**, reproduced below):

LMM3e: Signed KL divergence ~ Group * Preference * Self baseline impulsivity + Self baseline preference uncertainty + (1|ID)

After controlling for baseline preference uncertainty, our main interaction result remained significant: that is, the participants with mPFC damage were more influenced by impulsive relative to patient others, compared to healthy controls (group x others interaction HC vs mPFC: *b* [95% CI] = 0.28 [0.02 0.54], *p* = 0.034). However, the main effect for the lesion control group was no longer statistically significant (main effect LC vs mPFC: *b* [95% CI] = -0.36 [-0.73 0.02], *p* = 0.062), which suggests that the mPFC lesion group were not overall more susceptible to social influence compared to the lesion control group, after accounting for individual differences in preference uncertainty. We have added the results of this exploratory model to our supplement:

| **Table S6.** *LMM predicting susceptibility to social influence, with self baseline temporal impulsivity km as covariates (centred around the grand mean), controlling for self baseline preference uncertainty ku (centred around the grand mean)* | | | | |
| --- | --- | --- | --- | --- |
| **Fixed effect** | ***beta*** | **95% CI** | ***t*** | ***p*** |
| (Intercept) | 0.53 | [0.31 0.76] | 4.63 | <0.001 |
| Group (*HC* vs *mPFC*) | -0.21 | [-0.48 0.05] | -1.60 | 0.111 |
| Group (*LC* vs *mPFC*) | -0.36 | [-0.73 0.02] | -1.88 | 0.062 |
| Others (*patient* vs *impulsive*) | -0.21 | [-0.43 0.02] | -1.84 | 0.068 |
| Self baseline *km* | -0.09 | [-0.23 0.05] | -1.30 | 0.194 |
| Self baseline *ku* | -0.13 | [-0.33 0.08] | -1.20 | 0.232 |
| Group (*HC* vs *mPFC*) x Others | 0.28 | [0.02 0.54] | 2.13 | 0.034 |
| Group (*LC* vs *mPFC*) x Others | 0.05 | [-0.31 0.40] | 0.27 | 0.787 |
| Group (*HC*) x Self baseline *km* | 0.05 | [-0.11 0.21] | 0.57 | 0.567 |
| Group (*LC*) x Self baseline *km* | 0.09 | [-0.13 0.30] | 0.82 | 0.415 |
| Others x Self baseline *km* | 0.13 | [-0.01 0.26] | 1.85 | 0.065 |
| Group (*HC*) x Others x Self  baseline *km* | -0.08 | [-0.24 0.07] | -1.06 | 0.291 |
| Group (*LC*) x Others x Self  baseline *km* | -0.06 | [-0.27 0.15] | -0.61 | 0.545 |

Note. HC: healthy control group; mPFC: mPFC lesion group; LC: lesion control group; 95% CI: 95% confidence intervals. The mPFC lesion group is the reference group.

**R2.5**: Finally, I suggest that the authors consider revising the title so that it says "temporal discounting" instead of "temporal impulsivity." Impulsivity can refer to a number of different constructs, while "temporal discounting" more specifically refers to the phenomenon of overvaluing more immediate rewards at one's future expense.

**Response**: Thank you for your suggestion. We agree that ‘temporal discounting’ is a more specific term than ‘temporal impulsivity’. We have revised the title accordingly.

**Reviewer #3**: In this manuscript, Su and colleagues investigate the impact of prefrontal cortex lesions on individuals' temporal impulsivity and their sensitivity to social influence. The experimental design consists in a set of inter-temporal decision tasks, proposed to a relatively large cohort of lesioned patients, lesion controls and healthy controls. Participants alternate classical inter-temporal decision blocks, and blocks where they have to learn the temporal preferences of two other (simulated) people (one more impulsive and one more patient). The authors use Bayesian computational models to estimate baseline discounting and susceptibility to social influence, measured as the change in discounting after learning others' economic preferences. They then use structural neuroimaging (voxel-based lesion-symptom mapping) to link behavioral effects of lesions to specific area in the brain, and claim to demonstrate that the dmPFC is causally implicated in susceptibility to
social influence, with distinct ventral portions of mPFC involved in temporal discounting.
This manuscript represents a laudable effort to investigate the question of interest, with data collection on a relatively large cohort of rare patients, state of the art Bayesian models and statistical analyses, etc.

Nonetheless, I feel that severe experimental and analytical limitations weaken the solidity of the claims made in the current version of the manuscript. I hope the authors will find my comments useful to amend their claims.

**Response**: Thank you so much for your positive feedback on our work. We sincerely appreciate the time you invested in reviewing our work and providing valuable comments, which have greatly contributed to improving our manuscript.

**Main issues**
**R3.1**: Although I acknowledge that similar paradigms have been used in the past, I am not convinced at all that the current experimental paradigm allows to make solid claim on "social influence". Basically, the only "social" aspect of the task is the fake surnames displayed on the Others block. There is no non-social condition, such that it is entirely possible that the effects are purely driven by non-social instrumental associative learning.

**Response**: Thank you for your query. We agree that it is important to distinguish social and non-social processes in order to reveal the specificity of mechanisms studied. We would like to highlight several reasons why we firmly believe that this paradigm provides a robust framework for examining social influence. First, the manipulation of social influence and the veracity of the other participants was carefully planned and executed to ensure participants had the belief that the two others they learnt about reflected the real choices of two other people who had previously taken part in the experiment. We used a selection of common gender matched first names of the other two participants. The experimenter monitored carefully during the experimental session if any participant expressed disbelief in the nature of the two others, and we probed them with a debrief at the end of the experiment with open questions about whether they had any concerns or suspicions during the study. Secondly, participants clearly distinguished between impulsive and patient others in their behaviour. Healthy controls felt more similar to patient others, an effect now replicated across multiple studies (Moutoussis et al., 2016; Su et al., 2024; Thomas et al., 2022) and mPFC patients showed an asymmetry in influence from impulsive compared to patient others. Such a pattern of results would not be predicted if the same basic associative learning mechanism was driving effects. Thirdly, participants’ choices were incentivised. If their learning had been totally non-social they could have ignored the information and picked options in line with their actual temporal preferences since this directly impacted their payment at the end of the experiment. All these checks further demonstrated the validity of our task. Finally, a previous study that included a control condition featuring the same stimuli and actions, but without requiring discounting computations (Garvert et al., 2015), failed to produce changes in participants’ social discounting preferences. These results suggest that it is the simulation of others’ preferences that is crucial for the observed changes. This is a feature that is central to social decision-making (Boyd et al., 2011; Frith & Frith, 2023). Taken together, we are confident that our paradigm is a reliable approach for investigating social influence. Nevertheless, we agree that future research could include explicit non-social controls targeting different levels of explanation for social specificity (Lockwood et al., 2020).

**Discussion**:

*The question of whether the brain has specialised regions and circuits for social behaviour is central to social neuroscience^8,83–85^. Previous work has identified how social specificity may be realised at different levels of explanation^24^. Our task had many features to enhance its ability to capture social processes, including two different social others with different preferences, informing participants the choices they observed were from real others, and carefully probing for any disbelief in the social manipulation. Furthermore, existing studies including control conditions that match the same stimuli and actions, but do not require social simulation, have highlighted the importance of the social component of the influence effect^27^. Future studies could consider including a non-social control targeting different levels of explanation for social specificity to reveal the cognitive boundaries and specific neural systems that underpin social influence and whether they are common or distinct from non-social processes.*

**R3.2**: Although the task is elegant and sophisticated in the way it tries to create individual specific condition, based on individual estimated discounting rate, this also create a fundamental problem of comparability. Basically, given that the different group (lesion, lesion control and healthy controls) have different discount rates, they then face different social-influence/learning tasks, with different Others, such that isolating the effect of Groups on social influence seems virtually impossible. Maybe developping a model that explicit model the learning could start addressing this issue.

**Response**: Thank you for this query. We ensured that all participants had learning accuracy of others’ preferences well above chance in all groups, this meant that others’ preferences could indeed be accurately learnt so that susceptibility to social influence could be quantified. Secondly, our statistical models explicitly account for individual differences in baseline temporal impulsivity, and we show that all effects are robust to accounting for this individual variability (see results). Therefore, we disagree that isolating the effects of groups on social influence is virtually impossible. Thirdly, the mPFC group and lesion control group did not differ in their baseline discount rates, but did differ in how they were affected by social influence (see results). Finally, our lesion analysis showing dmPFC damage effects on social influence is within the lesion groups only and clearly demonstrates the effects of dmPFC damage on social influence, further corroborating the group-level analysis.

**R3.3**: L 255-257: one of the main behavioral result is a close to threshold (p = 0.031) interaction, arising from a complex mixed-model modelling factorial effects on computational parameters. How robust is this effect to different specifications of the LLM (e.g. including random slopes, or different set of covariates)?

**Response**: Thank you for your query. Our behavioural result is indeed very robust to different specifications of the LMM including a new analysis controlling for self baseline preference uncertainty (**Table S6**) as well as already presented analyses such as including depression and apathy scores (**Table S3**) and the order of others’ preferences (**Table S4**). We also showed no correlations with learning accuracy or self-reported similarity. Together these findings indicate that our analyses are robust despite to different specifications.

Although our sample size is large compared to many existing lesion studies, it is still limited by the participants available to test, and we tested a rare patient group, so cannot increase our sample size indefinitely. Despite this, we still used properly controlled quantitative statistics throughout and would abstain from qualitatively commenting on the numerical value of significance when our statistical threshold was a conventional *p* < 0.05.

Since each of our participants only has one data point per condition, we are unable to include random slopes in our mixed-effects models due to the lack of within-participant variation. For this reason, we did not include any random slopes for different others’ preferences, but we did include random intercepts to account for differences between participants. We have added these details to our supplementary methods:

***Supplementary Methods***

***Statistical analysis:***

*Note that each participant contributed a single parameter data-point and therefore these models could not contain random slopes.*

**R3.4**: A significant portion of the neuroimaging results are based on what seems to be extremely lenient statistical threshold (P< 0.05 uncorrected). While I acknowledge that the few reported voxels land in regions "of interest", I do not feel that these results build on solid statistical evidence, and I even more strongly urge against the idea of reporting them as one of the main claim/finding in the abstract.

**Response**: All lesion-symptom mapping (not neuroimaging) was based on standard statistical thresholds that appropriately handle multiple comparisons. To use an approach that appropriately balanced between type I and type II errors we used permutation testing combined with threshold-free cluster enhancement (TFCE), a method that effectively balances sensitivity to true effects while minimising the power to detect small, potentially spurious effects (Manohar & Husain, 2016). The use of permutation testing to control for multiple comparisons is widely regarded as the current ‘gold standard’ for correction in VLSM studies (Mirman et al., 2018). Therefore, the ‘*p* < 0.05 uncorrected after permutation-based TFCE’ is not a lenient statistical threshold. On the contrary, we applied a highly stringent criterion *a priori* by further applying Bonferroni correction after performing permutation testing with TFCE. Finally, we are transparent in acknowledging that the findings of damage to vmPFC and temporal impulsivity are exploratory. Despite this, they may still be of interest to readers and provide valuable insights for future research.

We have changed the wording in the results to avoid misunderstanding. We have also added further details about our lesion mapping approach to our results and methods:

***Results****:*

*Next, we used voxel-based lesion-symptom mapping (VLSM) to examine whether subregions within mPFC were linked to group differences in susceptibility to social influence. The VLSM analysis pinpoints voxels where participants with damage at that voxel, compared to participants with damage elsewhere, show differences in susceptibility to impulsive relative to patient social influence (i.e., signed impulsive D_KL_ minus signed patient D_KL_; N = 26 where both patient and impulsive others were present, see Methods). VLSM assesses whether the lesion in each voxel predicts an individual’s behaviour by generating a map of the t-statistics^53^. We included voxels where damage was present in at least five participants^54^. We used the FMRIB Software Library (FSL)^55^ to conduct permutation-based VLSM with threshold-free cluster enhancement (TFCE)^56,57^. The combination of permutation testing with TFCE allowed us to achieve an optimal balance between sensitivity to true effects and reducing the risk of identifying small, potentially spurious effects (Manohar & Husain, 2016). Significance was reported at permutation-based TFCE p < 0.025 (permutation-based TFCE p < 0.05 Bonferroni-corrected across two behavioural regressors). As a control analysis we first confirmed that there was no significant association between the overall degree of damage (i.e., total lesion size) and susceptibility to social influence (see Methods).*

***Figure 5 legend****:*

*(a)* *Permutation-based, whole-brain, non-parametric voxel-based lesion-symptom mapping (VLSM) showed that damage to dorsomedial prefrontal cortex (dmPFC, area 9) was associated with heightened susceptibility to impulsive relative to patient social influence (permutation-based threshold free cluster enhancement (TFCE) p < 0.025).*

***Figure 6 legend****:*

*(a) Permutation-based, whole-brain, non-parametric voxel-based lesion-symptom mapping (VLSM) showed that the areas 13 and 25 in the vmPFC as well as ventral striatum where damage was correlated with increased temporal impulsivity (permutation-based threshold free cluster enhancement (TFCE) p < 0.05).*

***Methods****:*

*We utilised FSL^55^ (v6.0.7.6)’s randomise function to conduct a permutation-based VLSM analysis^56,57^, which compares lesion participants with damage at each voxel to all other lesion participants. FSL has been validated for performing VLBM analyses and is widely utilised, as highlighted by its adoption in several recent lesion studies^125–128^. FSL implements the latest advancements in brain-based analysis, maintaining regular updates, and remaining open source. FSL also supports the use of threshold-free cluster enhancement (TFCE), which maximises power and uses non-arbitrary definitions of cluster size^56^. This feature is not currently available in other lesion-mapping toolboxes, such as LESYMAP and NiiStat. To increase power, we mirrored the lesion participants’ lesion maps, as we did not have specific hypotheses about laterality of mPFC function^120^, resulting in symmetrical masks. Voxels were included in the VLSM analysis only if at least five participants had some degree of damage in that voxel. Each behavioural regressor of interest was ranked to correct for skewness in the residuals distribution^57^ and then z-scored, in accordance with the requirements of FSL to align with the nature of our experimental design, before being input into the FSL design files.*

*P values were generated through permutation-based TFCE in randomise with 5,000 permutations and FSL’s default TFCE settings, which are optimised for this type of data^56,57^. Permutation testing repeats the same analysis multiple times with the randomly shuffled data to calculate voxel-wise P values, which estimate the probability that the observed effect could be attributed to random noise. This approach therefore more accurately reflects the nature of the data, relies on fewer assumptions compared to other methods, and can be combined with the advantages of TFCE^57^. Permutation testing is widely recognized as the ‘gold standard’ for addressing multiple comparisons in VLSM studies^129^. By combining permutation testing with TFCE, we effectively balanced sensitivity to true effects while minimizing the likelihood of detecting small, potentially spurious effects^53^. To ensure even greater stringency, we further applied a Bonferroni correction for multiple comparisons across the two behavioural regressors of interest (p < 0.025) to the uncorrected maps from the permutation-based TFCE results. For the purpose of visualisation, we applied binarized masks of the significant areas from each analysis to the t-values.*
